# Therapeutic Relational Connection in Telehealth: Concept Analysis

**DOI:** 10.2196/43303

**Published:** 2023-06-22

**Authors:** Lisa V Duffy, Rebecka Evans, Veronica Bennett, Joan Marie Hady, Priya Palaniappan

**Affiliations:** 1 School of Nursing Bouvé College of Health Sciences Northeastern University Boston, MA United States; 2 Bouvé College of Health Sciences Northeastern University Boston, MA United States

**Keywords:** therapeutic relational connection, telehealth, telemedicine, eHealth, telemedicine, concept analysis, provider-patient relationship, therapeutic relationship, relationship, connection, patient-provider, patient-physician

## Abstract

**Background:**

Therapeutic relational connection (TRC) in telehealth is a new concept that refers to the intentional use of relationship connection between health care providers and their patients as both parties work toward a therapeutic aim. It has been demonstrated that TRC positively affects patient-centered outcomes including adherence, self-management, and satisfaction with care. What is not known are best practices for establishing TRC during telehealth visits. The rapid emergence of telehealth during the COVID-19 pandemic has identified a number of challenges. These challenges include lack of human contact, distance creating mistrust, the inability to rely on nonverbal communication, and a sense of depersonalization. Training for health care providers in these interpersonal communication skills needed to establish TRC during telehealth visits is needed.

**Objective:**

This paper aims to explore the evolutionary concept of TRC in telehealth. The purpose of this paper is to provide a concept analysis of TRC during telehealth interactions between providers and patients through a comprehensive review of the existing published literature.

**Methods:**

Rodgers’ evolutionary concept analysis method was used to guide this study. PubMed, Embase, PsycINFO, and CINAHL were used to search for relevant publications. An integrative review strategy aided by Rayyan software was used to identify a final sample of 13 papers for analysis.

**Results:**

The proposed definition of TRC in telehealth is the experience of a mutually responsive patient-provider relationship that is built on mutual respect and understanding and informed by cultural humility, presence, empathy, and the ability to effectively evaluate patient concerns to work toward a therapeutic aim. The key attributes of TRC in telehealth are the provider’s ability to evaluate patient concerns, interpersonal communication, cultural humility, mutual trust and respect, presence, empathy, and building relationships. Clinical presence, proper environment, knowledge about the use of technology (both patient and provider), use of verbal and nonverbal communication, and knowledge about community and culture are important antecedents of TRC. Consequences of TRC include improved communication resulting in mutual respect and caring, adherence to follow-up recommendations, increased coping, collaborative decision-making, and satisfaction with care.

**Conclusions:**

Telehealth visits necessitate alternative approaches to establishing TRC as compared to in-person clinic visits. With the rapid expansion of telehealth platforms and a heightened acceptance of the technology, there is a need to integrate knowledge and provide a clear conceptualization of TRC in telehealth as TRC has been demonstrated to result in positive patient-centered outcomes. Identifying the attributes and antecedents of TRC in telehealth allows us the opportunity to develop guidelines and educational interventions aimed at training health care providers in the skills needed to establish TRC during telehealth visits.

## Introduction

Therapeutic relational connection (TRC) is a novel concept that refers to the intentional use of relationship connection between health care providers and their patients toward a therapeutic aim. The importance of patient-provider relationships has long been recognized in both the research literature as well as clinical practice. Specifically, it is the quality of the relationship between provider and patient that has been shown to positively affect clinical care outcomes including self-efficacy, adherence, morbidity, functional status, and satisfaction with care [1,2]. Relationship quality may be determined by the degree of connectedness in the context of patient-provider relationships. Researchers have found that patients’ perceptions of the extent of their connectedness or the quality of the relationship with their health care provider is associated with increased participation in medical decision-making and decreased risk-taking behaviors [2]. TRC is an essential part of working toward patient-centered outcomes.

TRC between providers and patients is developed through mutual feelings of trust, respect, caring, and actively including patients while making treatment decisions [3,4]. Small investments toward establishing TRC between patients and providers can make patients feel comfortable and empowered to discuss their health care needs [5]. Establishing TRC between patients and providers may be even more difficult because, since the start of the COVID-19 pandemic, a growing amount of health care has been provided via telehealth [6]. What is known is that during telehealth visits, health care providers dominate the conversation, have difficulty interpreting patients’ facial expressions, find the environment distracting, and find it more difficult to have patients adhere to treatment recommendations [7]. Patients have reported that during telehealth visits, their providers do not pay as much attention to them, they need to ask more clarifying questions, and being in separate spaces decreases the feeling of being connected to their provider [7]. What is unknown is how patient-provider relationships have been affected by the increased use of telehealth.

The Health Resources Services Administration [8] defined telehealth as “the use of electronic information and telecommunications technologies to support long-distance clinical health care, patient and professional health-related education, public health and health administration.” Technologies used to connect with patients include but are not limited to telephone contact, text-based messaging, videoconferencing, and wearable or implantable sensors and devices. Attention to the importance of TRC has been heightened by the COVID-19 pandemic where health care providers suddenly found themselves distanced from their patients and needing to connect via a variety of telehealth platforms [8]. In 2020, the use of telehealth increased by 50% in the first quarter as compared to the first quarter of 2019 [9]. The Department of Health and Human Services reported 52.7 million Medicare visits conducted via telehealth in 2020, which increased from 840,000 in 2019 [10]. Historically, research regarding patient-provider relationships has been centered on in-person interactions with limited data regarding how TRC is defined and established via telehealth visits [2,11]. TRC that is typically established during in-person visits can be interrupted during telehealth visits due to a variety of issues including faulty internet connections and environmental disruptions [12-14]. Some studies have found that telehealth can reduce patient participation in decision-making and reduce rapport [15]. The use of telehealth eliminates physical presence, and therefore, there are fewer ways to assess the patient, and the provider and patient have fewer cues to demonstrate empathy and understanding [12]. TRC can also be impacted as patients may perceive that the provider is not fully attending to them during a visit, they may feel rushed or find it difficult to speak up during an appointment [13]. When a patient feels that their provider has not been thorough, it can lessen TRC [12]. Telehealth platforms will continue to be a common way to deliver care in the future, with growing evidence showing effectiveness in the management of chronic illness [16]. With these new realities of health care delivery, it is essential to understand the best practices for establishing TRC during telehealth visits to ensure better patient-centered outcomes.

The purpose of this paper is to provide an evolutionary analysis of the novel concept of TRC in telehealth interactions between providers and patients through an evaluation of the published literature. The aim of this evolutionary concept analysis was to explore how patient-provider relationships are affected by the use of telehealth. The development of this novel concept is necessary as existing conceptual and theoretical frameworks do not address the intentionality of fostering mutually beneficial patient-provider relationships via telehealth platforms. TRC is influenced by relational cultural theory, which highlights the importance of mutuality to establish a growth relationship. Relational cultural theory is a humanistic theory that emphasizes the importance of positive relationships for personal growth over one’s lifetime [17]. Relational cultural theory posits that people grow through mutual empowerment and mutual empathy within community and relationship rather than by individual experiences [18]. Relational cultural theory also recognizes that sociocultural differences strongly impact relationship development [17]. When mutuality is fostered in relationships, there can be positive outcomes that include a sense of zest, relationship clarity, a sense of self-worth, creativity and productivity, and a desire for more connection [18]. These outcomes can help a patient achieve their health care goals by working together with their provider toward a therapeutic aim. Relational cultural theory adds to existing theories on telehealth as current theories do not address the intentional use of relationships to achieve mutually beneficial relationships. Evolving current theories by adding specific attention to the development of relationships adds a unique conceptual understanding of TRC.

Understanding how TRC is experienced and fostered during both synchronous and asynchronous telehealth interactions will help guide future research on improving patient-provider relationships through the development of guidelines and educational interventions to improve TRC during telehealth visits, which is ultimately improving patient-centered outcomes.

## Methods

### Approach

Rodgers’ evolutionary concept analysis method was used to guide the development of the concept of TRC in telehealth [19]. Conducting a concept analysis through Rodgers’ guidelines includes identifying the concept, alternative terms, and appropriate methods for data collection; collecting data to identify attributes, antecedents, and consequences; and providing an example and finding areas for further development of the concept [20]. In this method, attributes refer to the cluster of characteristics comprising the definition of the concept. Antecedents are events that have been associated with the concept, and consequences are what happens as a result of the concept. Rodgers’ evolutionary concept analysis method acknowledges that concepts shift over time, influenced by use, application, and significance, resulting in an analysis that is practice-related and current [21]. Concept analysis is a key component of knowledge base development and enables systematic and cohesive understanding of the concept being investigated [22]. It is through understanding the conceptual development and current state of the science related to the concept of TRC in telehealth that we can link conceptual work to solution-based scholarship.

### Search Strategy

An integrative review strategy as outlined by Whittemore and Knafl [23] was used to methodically search the existing literature on phenomenon related to TRC in telehealth. PubMed, Embase, PsycINFO, and CINAHL were used to search for publications. The keywords used were “patient provider relationship,” “physician patient relation,” “nurse patient relations,” “patient provider connection,” “connectedness,” “provider rapport,” “nurse-patient relations,” “telehealth,” “telemedicine,” “remote,” “virtual,” and “virtual visits.” The initial search resulted in 2763 papers. Rayyan software (Rayyan Systems, Inc) was used to systematically review papers through a blinded collaborator methodology. The inclusion and exclusion criteria can be found in Textbox 1.

**Figure 1:**
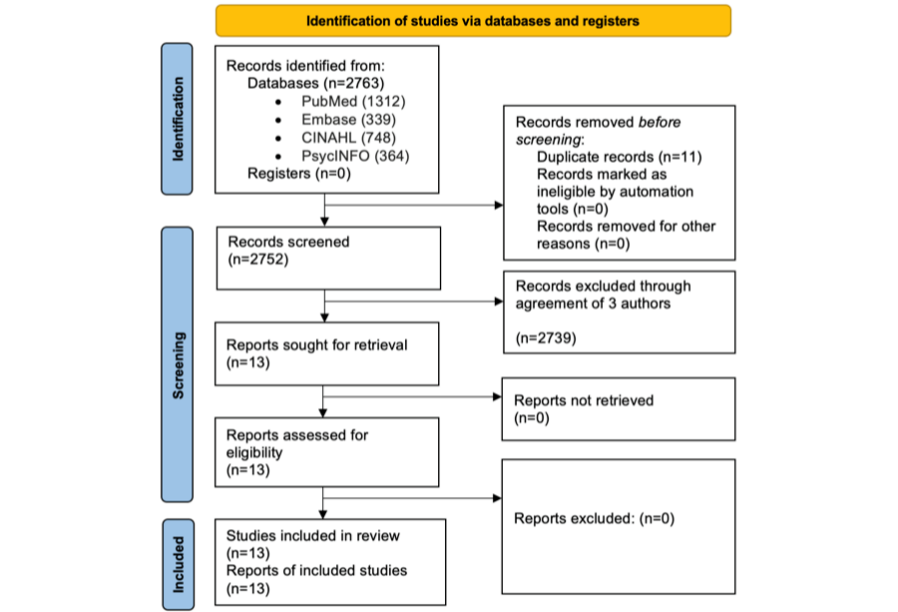
PRISMA (Preferred Reporting Items for Systematic Reviews and Meta-Analyses) flow diagram of selected studies for review.

Each title was reviewed to identify papers that reflected the concept of TRC, and papers were excluded that did not meet these criteria. Next, the remaining abstracts were reviewed to determine if inclusion criteria were met. Papers meeting inclusion criteria were read in their entirety, and those that did not reflect the concept of TRC in telehealth were excluded. As the final step for paper inclusion, collaborators were unblinded, and any conflicts around inclusion or exclusion of papers were resolved through open discussion. In total, 13 papers were included in the final concept analysis (Figure 1).

In total, 9 papers described studies using qualitative methodology [13-15,24-29], and 4 were systematic or literature reviews [12,30-32]. The modes of telehealth used in these papers include videoconferencing [12-15,24-32], using audio only (over the telephone or internet) [12,14,15,28,31], text-based messaging [12,14,28,29,31], asynchronous messaging (video or audio) [12,14,26-28,31], and remote data management [12,14,15,26-28,31].

**Inclusion and exclusion criteria for search strategy.**

**Inclusion criteria**

- Paper type
  Published within the last 10 years
- Language
  Discusses patient-provider relationships during telehealth encounters

**Exclusion criteria**

- Paper type
  Nonresearch-based studies
- Language
  Non-English papers
  Discusses patient-provider relationships in an in-person or clinic setting

## Results

### Overview

There is currently no standard definition of TRC in telehealth and the literature references several different terms for this phenomenon. In total, 10 of the 13 papers reviewed identified the importance of patient-provider relationships in understanding connection or alliance. Terms used to refer to the concept of TRC in the reviewed papers were doctor-patient relationship [27,29], patient-provider relationship [13-15], doctor-patient interaction [12,31], and nurse-patient relationship [24]. Connection was identified as a therapeutic relationship or alliance [15,30,32], connection [25,26], rapport [13], closeness [31], socioemotional communication [29], and empathic communication or affective expression [14]. The term presence, defined by Tuxbury [28] as the reciprocal flow of openness, and dialog was used as a surrogate for TRC and was described in 3 papers [24,28,30] (Table 1).

Included papers were reviewed to determine the attributes, antecedents, and consequences of TRC in telehealth. Table 2 summarizes each paper’s contribution to the conceptual elements of TRC in telehealth.

**Table 1:** Surrogate terms for therapeutic relational connection.

Paper	Author	Year	Surrogate or similar terms
1	Hiratsuka et al [26]	2013	Connection, relationship or nonverbal communication
2	Tuxbury [28]	2013	Presence, reciprocal flow of openness and dialogue
3	Barrett [24]	2016	Presence or nurturing presence, nurse-patient relationship
4	Goldstein and Glueck [32]	2016	Therapeutic alliance or developing rapport
5	Henry et al [31]	2016	Communication, closeness or building rapport, fostering collaboration, interpersonal behavior and attitudes, provider-patient interaction
6	Piras and Miele [27]	2019	Patient-provider relationship or digital intimacy
7	Chua et al [25]	2020	Feeling of connection or web-side manner
8	Gordon et al [13]	2020	Therapeutic rapport, provider-patient relationship or communication, web-side manner
9	Yan et al [29]	2020	Doctor-patient relationship, socioeconomic communication or understand and respond to patient emotional response, computer-mediated communication
10	Botrugno [12]	2021	Doctor-patient interaction or social presence, being there
11	Cataldo et al [30]	2021	Presence, therapeutic relationship or therapeutic alliance, computer-mediated communication
12	Kludacz-Alessandri et al [15]	2021	Provider-patient relationship, therapeutic relationship or interpersonal communication, relationship, respect
13	Zhang [14]	2021	Patient-provider relationship or doctor-patient communication, empathetic response, clinical empathy

**Table 2:** Papers informing conceptual elements.

Concept element			Papers
**Antecedents**			
	Confidence in clinical abilities	Gordon et al [13] Barrett [24]	
	Suitable environment	Gordon et al [13] Barrett [24] Henry et al [31]	
	Cultural humility	Hiratsuka et al [26]	
**Attributes**			
	Evaluate concerns	Barrett [24] Henry et al [31]	
	Communication	Kludacz-Alessandri et al [15] Barrett [24] Henry et al [31]	
	Cultural humility	Hiratsuka et al [26]	
	Trust and respect	Gordon et al [13] Zhang [14] Kludacz-Alessandri et al [15] Barrett [24] Hiratsuka et al [26] Piras and Miele [27] Yan et al [29] Cataldo et al [30] Henry et al [31] Goldstein and Glueck [32]	
	Presence	Barrett [24] Tuxbury [28] Cataldo et al [30] Henry et al [31] Goldstein and Glueck [32]	
	Bidirectional responsiveness	Botrugno [12] Henry et al [31]	
	Empathy	Botrugno [12] Zhang [14] Barrett [24] Chua et al [25] Goldstein and Glueck [32]	
	Relationship	Botrugno [12] Hiratsuka et al [26]	
**Consequences**			
	Improved communication	Gordon et al [13] Barrett [24] Chua et al [25] Yan et al [29] Henry et al [31] Goldstein and Glueck [32]	
	Coping	Hiratsuka et al [26]	
	Decision-making	Zhang [14] Barrett [24] Chua et al [25] Cataldo et al [30] Goldstein and Glueck [32] Henry et al [31]	
	Satisfaction with care	Zhang [14] Barrett [24] Chua et al [25] Cataldo et al [30] Henry et al [31] Goldstein and Glueck [32]	
	Diagnosis	Zhang [14] Kludacz-Alessandri et al [15] Barrett [24] Chua et al [25] Yan et al [29] Henry et al [31] Goldstein and Glueck [32]	
	Adherence	Kludacz-Alessandri et al [15] Chua et al [25] Cataldo et al [30] Henry et al [31]	

### Attributes of TRC

The defining attributes of TRC in this concept analysis were identified as “provider ability to evaluate patient concerns,” “interpersonal communication skills,” “knowledge of patient community and culture,” “mutual trust and respect,” “presence,” “bidirectional responsiveness,” “empathy,” and “relationship.” Each of these attributes will be further described in detail.

#### Provider Ability to Evaluate Patient Concerns

To facilitate TRC in telehealth, health care providers must accurately evaluate patient’s concerns. The nature of telehealth visits removes the health care provider’s ability to complete a hands-on examination, and they therefore must use alternative methods, namely, communication to assess patient needs by obtaining a detailed clinical history. Health care providers rely on positive relationships to engage patients to obtain this quality clinical data [31]. Barrett [24] described the ability to evaluate the patient’s concerns via telehealth as part of clinical presence. Evaluating patient’s concerns requires communication as a vital skill that contributes to the development of TRC.

#### Interpersonal Communication Skills

Interpersonal communication skills include the ability to assess patients’ verbal and nonverbal cues, including understanding emotional expressions and feelings and responding to these cues with empathy. Verbal communication includes behavioral skills, timing, clinical conversation, and nonverbal communication includes eye contact, visual cues, and empathetic gestures [31]. When patients feel heard, they are better able to communicate concerns and respond with clarification and details [5]. Health care providers’ ability to listen was assessed by patients as extremely important in most of the papers reviewed as this attribute conveys connection and presence to patients. Patients appreciate active listening, the ability to explain health issues and engage in collaboration [15]. There is a need for bidirectional responsiveness with interpersonal communication where both the patient and the provider rely on cues from the other to communicate [31]. Bidirectional responsiveness within telehealth provides the opportunity for real-time feedback and adaptivity for both the patient and provider as is common during in-person communication.

#### Knowledge of Community

Health care providers need to have sociocultural understanding of the community they are serving [26]. This includes a broad understanding of the culture of the community being served, including social roles and interactions between community members as well as an understanding of specific community practices. Health care providers also reflect on the need to avoid assumptions and seek clarification about culture and community [26]. Hiratsuka and colleagues [26] found that cultural awareness and sensitivity along with demonstrated respect and caring were more important than the specifics of a telehealth visit.

There can be significant disparities in access to telehealth with higher usage among White, middle-aged, English-speaking patients in urban settings, those with more broadband availability as well as those with health insurance and higher incomes [33]. This knowledge of culture and community is an important attribute of TRC.

#### Mutual Trust and Respect

Most reviewed papers identify mutual trust and respect as factors integral to the development of TRC and resulting satisfaction with health care delivery [13-15,24,26,27,29-32]. Kludacz-Alessandri et al [15] reported that patients highly valued empathy and respect, seen as active listening, feeling heard, being patient, and careful. One way of promoting trust and respect is for the health care provider to have their professional qualifications available for the patient on associated health care websites [14]. Information should include a profile photo, affiliations, education, background, specialization, and experience [14]. Zhang [14] explores the idea of commercialization of health care, where patient satisfaction is prioritized in the delivery of care. As patients feel empowered in their decisions in choosing a provider, the providers also adapt to the needs of the patients, thus creating a relationship that is patient-centered. Regard for the environment is an additional aspect of mutual caring and respect. Environment is especially important for clinicians as patients feel safer when the telehealth environment looks familiar and has limited distractions [31].

#### Presence

Presence is the state or fact of being in a place or a state but also reflects an appearance or manner [24]. Barrett [24] explored this concept in depth and created subcategories of presence as operational, clinical, therapeutic, and social presence. When the health care provider demonstrates social presence, the patient (and provider) is more at ease [24]. For this concept analysis, presence is an attribute that characterizes TRC as it embodies overarching elements needed to establish TRC. The phenomenon of presence can be identified within several other attributes of TRC; however, it also stands independently, as it reflects the roles played by both the provider and the patient. Both must be involved in the interaction and willing to take part, and for TRC to occur, there must also be active engagement in the interaction [32]. When presence is lacking, the overall relationship is impacted and so is the effectiveness of the treatment [30]. Telehealth may necessitate the development of a “video presence” with exaggerated facial expressions, awareness of room, surroundings, eye contact, and body language [31].

#### Building Relationships

Providers and patients recognize the importance of building relationships to have an effective and satisfactory interaction [26]. This can be considered a part of other attributes of TRC; however, it is independently identified in most of the papers reviewed. Part of building relationships includes effective communication, cultural awareness, mutual respect, and caring. Patients identified feeling more connected when they felt their health care provider was caring, listened, clarified information, collaborated, and was competent [26]. Relationships between providers and patients develop over time in telehealth visits, with the provider needing to adapt their behaviors and interactions so the patient feels secure and connected [12]. While there is a perceived risk of telehealth dehumanizing patient-provider relationships [12], sustained effort to understand this innovative technology can consider the shifts in communication and experiences to develop relationships in TRC.

#### Empathy

The Merriam-Webster dictionary defines empathy as “the action of understanding, being aware of, being sensitive to, and vicariously experiencing the feelings, thoughts, and experience of another of either the past or present without having the feelings, thoughts, and experience fully communicated in an objectively explicit manner” and as “the capacity for having empathy” [34]. Empathetic communication is an essential attribute of TRC. Patients report more satisfaction with telehealth when they feel the provider is empathic [14]. Patients look to providers to be a relatable resource for support and empathy [12]. Zhang’s [14] review of the phenomenon of empathic communication in telehealth identified the major components of empathic responses and affective expressions. Empathic communication is comprised of patients’ implicit and explicit expression of negative emotions or feelings and providers’ empathic responses (expressions) that explicitly recognize patient affect and emotional states [14]. In Barrett’s [24] description of therapeutic presence, he noted that nurses can recognize and respond to nonverbal cues and offer comfort and reassurance to patients. The use of simple and complex reflections (by the provider) is the way of demonstrating empathy and building connection [25]. Goldstein and Glueck [32] noted the importance of conveying empathy through fluid responses to patients and families. When patients feel the emotional tone of a response and providers can convey understanding, patients have better experiences [25]. Acting with empathy shows patients that the provider is making a committed effort to provide thoughtful clinical care [12]. This affective communication and conveyance of empathy are important in the development of TRC.

### Antecedents of TRC

Antecedents are the events that need to be present prior to the development of TRC [21]. In this analysis, antecedents were categorized as confidence in clinical abilities, suitable environment, and knowledge of community.

#### Confidence in Clinical Abilities

The provider needs the ability to clinically evaluate the concerns of the patient and treat them effectively. Providers reported feeling concerned they might miss an important piece of information not being in the same room as the patient [26]. Patients are concerned that quality of care may be diminished in telehealth visits because providers are unable to carry out a physical examination or because they do not have enough information [13]. If a patient perceives that the provider is uncertain or limited, the patient becomes uncomfortable [13]. Given these concerns and the reality of the telehealth visit, it is critical for TRC that providers be able to effectively demonstrate clinical abilities. Barrett [24] described clinical presence, which includes history-taking, visual examination, and conveyance of treatment recommendations. For patients to find connection and comfort, they need to be able to supply data (subjective and objective) and know that the provider can effectively use that data to care for them.

#### Suitable Environment

A suitable environment can facilitate interactions, allow for the recognition of verbal and nonverbal cues, limit distractions, and is accessible to both the provider and the patient [31]. In terms of telehealth medical visits, this means that appointment structure and content are reproducible, the participants have knowledge around use of the technology platform, the participants are willing to adapt to the telehealth visit experience, and that the technology is accessible and consistent. An example of this is a private, well-lit setting, maintaining eye contact between provider and patient or caregiver and providing proper orientation to the specific telehealth format [25].

#### Cultural Humility

Open-minded attitudes and cultural competency help patients feel more comfortable and confident in their providers and allow for mutual respect and collaborative decision-making. When patients feel they are understood and that their concerns are being addressed, therapeutic alliance increases. Cultural competence can also result in positive attitudes toward telehealth [31]. In some communities, such as in the Native Hawaiian and Alaska Native populations studied by Hiratsuka et al [26], there is an increased likelihood that the providers and patients will have dissident cultures and backgrounds. Awareness and attention to this element can help facilitate patient-provider relationship and foster TRC. Providing culturally appropriate care is one way to demonstrate respect for your patient and their family [26].

### Consequences of TRC

**Figure 2:**
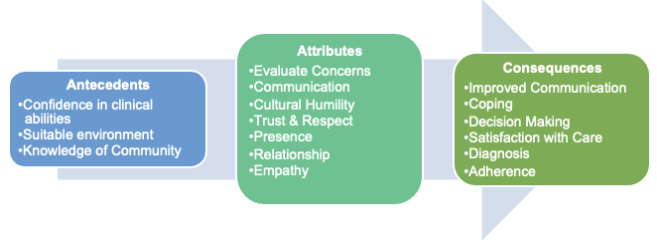
Process in development toward therapeutic relational connection.

TRC is associated with several positive outcomes. An overarching consequence is that TRC improves the quantity and quality of patient-provider communication, which increases mutual respect and caring, benefiting both providers and patients. TRC allows providers to obtain quality information from patients, resulting in improved well-being and a sense of purpose. For patients, TRC results in an increased commitment to follow-up [26,28], feeling emotionally supported [2], and an increased ability to cope [26,28]. For both providers and patients, there is an alliance [24,25,31,32], improved communication (quantity and quality) [14,28-31], mutual respect and caring [25,29], enhanced relationship [14,24,29,31,32], promotes long-term partnership [14,24,30], collaborative decision-making [31,32], greater satisfaction with care [29], patient and provider more at ease [24,25,30], empathy [2,14,24,32], more accurate diagnosis of problems [14,31], and being present [24,31,32]. For patients, there is improved follow-up care [9,15], feeling emotionally supported [15,25,28,29,31], improved health outcomes [9,15,31], improved adherence to treatment plan [9,31], patient satisfaction [15,28], feeling heard and understood [15,25,28,30], and empowerment [15,25]. Providers gain a sense of well-being and sense of purpose [1,14,24,28], improved assessment of the patient [1,14,29], increased familiarity with patients [28,31], and understanding of patient concerns [1,14,29,31] (Figure 2).

### Empirical Referents

Empirical referents are ways in which we see TRC demonstrated in telehealth. One example of how this concept is demonstrated was by Hiratsuka et al [26] who found that when providers engaged with Alaska Native and Native Hawaiian people recognized language barriers and took the time to make sure that the information was understood, patients felt more comfortable with the providers’ willingness to listen and showed them respect and caring. Botrugno [12] gave examples of how the behaviors of providers and patients during consultation can create TRC. When patients feel more in control and there is more expression of emotions and discourse between patient and provider, the result is adherence to treatment plans of care and improved health outcomes. Barrett [24] described therapeutic presence as offering comfort and reassurance to patients through “being with the patient” and taking the time to make them comfortable; “they’re pleased to see you, often quite reassured, they like to hear your voice, see your expression” [24]. When patients have affective expressions and providers have empathic responses, TRC is established, more information is obtained leading to long-term partnership and better health outcomes [14]. Awareness of verbal and nonverbal communication conveys empathy and increases rapport; this can be done with a simple wave of hello to put the patient at ease [25]. To better illustrate the application of TRC in clinical practice, the following patient exemplar is presented.

### Exemplar Case

Andrea Marshall (pseudonym) is a young adult with a diagnosis of multiple sclerosis (MS). She lives in a rural community where she does not have access to a neurology provider specializing in MS. She has been referred to a clinic hundreds of miles away but will need to have a telehealth visit to establish care. Andrea Marshall has been prescribed a disease-modifying therapy from a local provider, but she has not been adherent with taking this medication. During her first videoconferencing visit with the MS specialist, the provider initiates the visit by introducing themselves and explaining how the visit will proceed. The provider ensures that the environment is suitable by talking with Andrea Marshall about lighting, placement of the camera, and decreasing distractions. The provider then takes time to find out more about Andrea Marshall. They have a casual conversation about her likes and dislikes, and the provider uses humor to put her at ease. During the visit, the provider learns the patient lives in a community where there is mistrust in health care providers and general disbelief in modern medicine. The provider takes the time to discuss the patient’s values and preferences around taking the disease-modifying therapy for her MS. The patient is interested in taking the medication, and both parties strategize how the patient may adhere to the prescribed regimen without feeling ostracized from the community. Throughout the visit, the provider carefully maintains eye contact and is intentional about their use of body language to convey empathy. This behavior allows the patient to feel comfortable, and they ask several questions. The provider conducts their telehealth examination and demonstrates competency by explaining what they are doing and why. The provider takes time to provide education on the new medication and the importance of close follow-up. The provider uses active listening throughout the visit. At the end of the visit, the provider ensures that all of the patient’s questions have been answered, and they schedule a follow-up meeting via videoconferencing in 2 weeks to review the treatment plan. The patient thanks the provider for their time and follows up after 2 weeks.

## Discussion

### Principal Findings

TRC is a novel concept. Although we have appreciated the importance of patient-provider connection in the past, little attention has been paid to the intentionality of TRC in the setting of telehealth. Intentionally creating a mutually beneficial relationship using telehealth platforms goes beyond what we currently think of as web-side manner or telepresence. Those concepts do not address the intentionality or bidirectional nature of patient-provider relationships affecting patient-centered outcomes. Recognizing that skills needed to establish TRC via telehealth are unique from those used during in-person visits allows providers to make necessary adjustments to their clinical approach. This concept allows us to better understand what skills are needed to achieve TRC in telehealth. TRC is the experience of a mutually responsive patient-provider relationship that is built on mutual respect and understanding. Based on the findings of this concept analysis, the proposed definition of TRC is the intentional use of relationship connection between health care providers and their patients toward a therapeutic aim.

Prior to the establishment of TRC, both providers and patients need to be comfortable with and confident in providing and receiving health care via telehealth. There continues to be hesitancy among both groups as providers often fear missing important clinical information [26] and patients are concerned their providers may miss important information as providers are often less attentive during telehealth visits [13]. Currently, there are no practice standards for training health care providers in telehealth, which may contribute to the lack of confidence using these platforms. Patients need to be assured they will receive the same care via telehealth that they would in-person, and providers can play a significant role in making their patients feel comfortable. Providers should be trained in performing physical examinations and taking clinical histories via telehealth to increase their confidence. Comfort for the patient can additionally be increased by ensuring the environment is conducive to a clinical visit. This should include a plan to address issues with technology and a way to minimize distractions and promote privacy. One example of how to establish trust via telehealth is to explicitly discuss issues concerning patient privacy [32]. Based on the concept analysis presented in this paper, there are many skills providers can adopt to improve the telehealth experience for both them and their patients.

Communication needs to be a central focus of establishing TRC in telehealth. It has been noted that conversations between patients and providers via telehealth are often dominated by physicians with less small talk and overall shorter visit length [31]. Providers need to be trained on the skills needed to successfully communicate with patients via telehealth platforms. This training should include enhanced interpersonal communication by verbally reflecting on what the patient is saying to improve the conversation [25]. Additionally, providers need to ensure their communication includes not only information but also emotion, which can help demonstrate empathy [12]. One example is responding to a patient fluidly without unnecessary breaks or pauses in the conversation helps to reinforce the demonstration of empathy [32]. Being empathetic in responses resulted in greater patient satisfaction and improved problem-solving [14,26]. During in-person visits, delayed pauses during conversation are more natural as you can interpret body language. During telehealth interactions, delayed responses, lack of initiative, lack of emotional comfort, and being unfriendly lead to patient dissatisfaction with the visit [29]. Patients want to feel that their provider is present, which is often more difficult to establish when both parties are not in the same physical space.

To establish presence, nonverbal communication is important [25,26]. When eye contact was lacking, patients reported feeling unheard and neglected by their providers [13]. Approach to communication differs depending on the type of telehealth platform. While eye contact has been demonstrated to help control communication [30], it is not an option for telehealth platforms that are asynchronous. In these situations, communication needs to convey essential elements of the relationship. Telehealth has significant potential to strengthen the bond between providers and their patients. Telehealth has been shown to provide a method for providing emotional support resulting in a deeper understanding of patients’ emotions and perceptions [27]; however, providers need more training as they perceive rapport to be lower when using telehealth [32]. Clinical presence might be inhibited by lack of touch [24]. One way to overcome this barrier is to increase the frequency of visits [24]. Innovative telehealth practices should consider the frequency of visits as it may compensate for the lack of in-person touch. Presence is reciprocal, and both patient and provider must choose to work toward a shared goal [28].

A crucial goal of TRC is working together toward achieving a therapeutic aim. Trust is necessary for patients to work toward common goals established between themselves and their providers; however, this often requires touch during in-person visits [30]. Without in-person touch, establishing trust requires more interactions over telehealth, and it takes longer to establish when compared to in-person visits [30]. This provides additional support for the need for more frequent telehealth interactions.

Bidirectional responsiveness is important to the development of TRC as both parties need to be invested and working toward the same outcomes. The bidirectional aspect of relationships evolves over the course of treatment [32]. Relationships were more difficult to establish via telehealth if the patient and provider did not have a prior relationship as small talk was found to be more difficult to initiate [13]. Providers need to approach new patients with more time realizing that it is more difficult to establish TRC with new patients when using telehealth.

Understanding TRC within the conceptual framework of relational cultural theory allows researchers and providers to design and implement strategies for training providers in the use of telehealth from a unique perspective. Future research should consider telehealth as a modality to improve TRC in vulnerable populations. Patient populations who rely on technology or those who cannot regularly access clinic visits in person could greatly benefit from developing relationships with their providers in a remote setting. Evidence demonstrates the benefit of TRC; however, we need to better establish practice guidelines for considering the intentional application of TRC in practice. There are countless opportunities to address disparities and improve equity by incorporating the intentionality of TRC in telehealth platforms.

### Limitations

There are several limitations to the methodology used in this concept analysis. As this paper aimed to explore a new concept, the search strategy may have unintentionally excluded additional terms that are associated with the elements of TRC. There was no empirical evidence to support our findings. Each of the papers reviewed in this paper was systematic review or used qualitative methodology. Telehealth platforms have only recently emerged as a widespread modality for medical appointments so the search strategy resulted in limited data regarding TRC in telehealth.

### Conclusions

Patient-provider connection and relationships have long been acknowledged as an important aspect of patient care. Past research on connection has explored connectedness and therapeutic relationships, but there has been limited exploration of how TRC is found and fostered in telehealth. This analysis supplies a preliminary theoretical framework of TRC. This framework will be useful in developing tools to measure TRC as well as evaluate and educate providers on how to improve their ability to connect to patients during telehealth appointments.

This analysis aimed to clarify the concept of TRC in telehealth. Telehealth necessitates alternative modes of connection and relationship development as compared to in-person clinic visits. With the rapid expansion of telehealth platforms, there is a need to provide a clear conceptualization of TRC in telehealth in order to establish best practices for health care providers. This work will enable the development of guidelines and educational interventions aimed at improving TRC in telehealth and guiding future research with the goal of optimizing patient-centered outcomes.

## Abbreviations

MS: multiple sclerosis
TRC: therapeutic relational connection
